# The Windy City Rookery: Movement and Activity Patterns of Black‐Crowned Night Herons (*Nycticorax nycticorax*) in a Human‐Dominated Landscape

**DOI:** 10.1002/ece3.73310

**Published:** 2026-03-31

**Authors:** Sarah Slayton, Henry Adams, Michael Avara, Brad Semel, Amy Lardner, Liza Lehrer, Michael Ward

**Affiliations:** ^1^ Department of Natural Resources and Environmental Science University of Illinois, Urbana‐Champaign Champaign Illinois USA; ^2^ Urban Wildlife Institute, Lincoln Park Zoo Chicago Illinois USA; ^3^ Illinois Department of Natural Resources Springfield Illinois USA; ^4^ Chicago Black‐Crowned Night Heron Project Chicago Illinois USA

**Keywords:** conservation, GPS/GSM tags, movement, urban, wading birds

## Abstract

As urbanization continues to accelerate, human‐dominated habitats are becoming increasingly important for wildlife as some species of conservation concern move into urban landscapes. In response to the widespread loss or conversion of their preferred wetland habitat, black‐crowned night herons ( *Nycticorax nycticorax* ; BCNH) frequently nest in urban areas. Despite being the most widely distributed colonial wading bird in the world, BCNH face population declines and have been listed as a special‐status species in 11 U.S. states and Ontario, Canada. The largest remaining BCNH rookery in the Great Lakes region of the U.S. is located at Lincoln Park Zoo in Chicago, Illinois. Although nesting in an urban center is inherently risky, this colony appears to be thriving. We used GPS/GSM satellite transmitters to better understand the home range size, habitat use, and activity patterns of individual birds during the breeding season. We found that BCNH foraged in a wide variety of natural and highly modified waterbodies and exhibited distinct behavioral differences between breeding and non‐breeding birds. Non‐breeding birds' home ranges were on average 18 times larger than those of breeding birds, which primarily foraged within 10 km of the colony, while non‐breeding birds ranged widely but continued to use relatively urban habitats. All birds were active throughout the 24‐h period, but breeding BCNH showed higher activity rates than non‐breeding birds during daytime hours. Understanding the behavior of this declining species within urban landscapes can improve our understanding of this species' ecology and provide valuable insights to inform management and conservation efforts.

## Graphical abstract

Black‐crowned night herons (BCNH) are a cryptic, widely distributed colonial wading bird. Despite showing regional declines in North America, this species has successfully adapted to colonize urban areas. In this study, our team used GPS/GSM transmitters to explore the space use, habitat use, and diel activity patterns of BCNH breeding at the largest remaining BCNH rookery in the U.S. Great Lakes region, located in the densely populated city of Chicago, Illinois, USA.

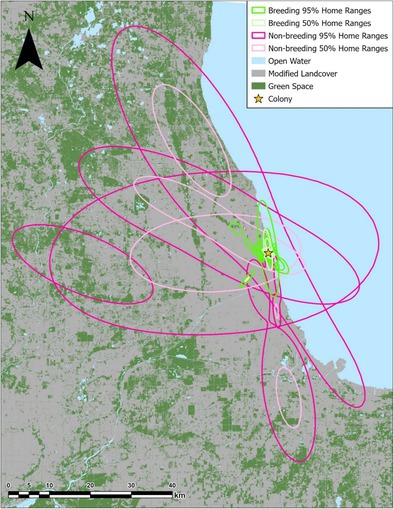

## 1 Introduction

Many birds move widely to acquire the resources necessary for survival and reproduction (Jones 2001; Kidd‐Weaver et al. 2020). These energetic demands shape avian movement patterns, habitat selection, and diel activity, as individuals must locate areas that provide sufficient food, shelter, and protection from predators (Jones 2001; Cooke 2019). This is especially true during the breeding season, when birds must efficiently forage across the landscape to meet their own nutritional needs and those of their chicks (Yurek et al. 2024). Understanding how birds select and move between habitats during the reproductive period is essential to understanding their ecology and informing effective conservation strategies, particularly for species of conservation concern breeding in human‐dominated landscapes (Haig et al. 1998; Fidorra et al. 2016).

Colonial wading birds (Ardeidae, Threskiornithidae, Ciconiidae) are highly mobile and known for their ability to locate and exploit patchy and unpredictable food resources (Kelly et al. 2006; Fidorra et al. 2016; Mott et al. 2023). These species rely on foraging habitats with shallow water where they can stand or walk while hunting for aquatic or semi‐aquatic prey, which often show rapidly fluctuating prey availability (Kushlan 1976; Burger 1981; Bernick 2004; Mott et al. 2023). Although wading birds may travel long distances in search of food during the non‐breeding season, individuals breeding in colonies typically forage within 10–20 km of their nesting site (Nemeth et al. 2005). As central‐place foragers, breeding birds are constrained in their habitat use and foraging range by the need to return to the colony multiple times per day to provision chicks (Orians and Pearson 1979; Lalla et al. 2022).

Historically, colonial wading birds foraged across shallow patches within wetland complexes or geographically isolated wetlands (Herteux et al. 2020; Mott et al. 2023). However, widespread habitat loss and degradation have caused many species to increasingly occupy human‐modified habitats such as cities (Taylor II et al. 2010; Fidorra et al. 2016; Hernandez et al. 2016; Kushlan 2018). Life in a highly altered landscape presents new challenges such as reduced breeding habitat, pollutants, and direct human disturbance (Parsons and Burger 1982; Kelly et al. 2006; Hunt 2016). Despite these drawbacks, these habitats may provide easier access to food resources for wading birds, potentially offsetting the disturbances associated with anthropogenic environments (Bernick 2007; Hernandez et al. 2016; Hunt 2016; Kidd‐Weaver et al. 2020). Urban wetlands may have more stable hydroperiods to support a more consistent food supply, vegetation‐free shorelines that facilitate easier hunting, enhanced prey productivity from nutrient runoff, and artificial light that may concentrate aquatic prey in water bodies (Dwyer et al. 2013; Maccarone and Hamilton 2014; Fidorra et al. 2016; Herteux et al. 2020).

Adaptation to urban life has also altered key behavioral and life‐history traits in several wading bird species, including geographic range size, breeding phenology, and migratory patterns (Partecke and Gwinner 2007; Shephard et al. 2015; Jennings et al. 2021). For example, urban populations of American White Ibis ( *Eudocimus albus* ) in southern Florida with access to year‐round anthropogenic food sources have transitioned from a nomadic to a residential lifestyle (Kidd‐Weaver et al. 2020) and show higher rates of habitat specialization and site fidelity during the non‐breeding season compared to their non‐urban counterparts (Teitelbaum et al. 2020). Similarly, some European White Storks ( *Ciconia ciconia* ) have changed their migration routes, wintering grounds, and stopover locations to exploit anthropogenic habitats such as landfills and agricultural areas (Shephard et al. 2015).

Urbanization can also influence an organism's diel activity patterns, or the distribution of its overall activity across the 24‐h period (Vallejo‐Vargas et al. 2022). While many species demonstrate rigid circadian rhythms that define their daily cycles of activity and rest, others show behavioral plasticity that allows them to adjust activity periods around local environmental conditions or shift them in response to seasonal energetic demands (Reed et al. 1999; Efrat et al. 2019; Tomotani et al. 2023). In urban environments, artificial light at night, direct human disturbance, and anthropogenic noise have been shown to alter how animals distribute their activity during the day and night (Isaksson et al. 2018; Morelli et al. 2018; Tomotani et al. 2023). For example, many urban mesocarnivores have shown increases in nocturnality to avoid peak hours of human activity, which in turn have increased daytime activity in their prey (Gallo et al. 2022). Such temporal shifts can affect foraging and reproductive activities but may also confer adaptive advantages for flexible species, particularly in urban settings (Titulaer et al. 2012; Mason et al. 2016; Gryz and Krauze‐Gryz 2019).

The black‐crowned night heron ( *Nycticorax nycticorax* ; BCNH) is a species of colonial wading bird that has successfully adapted to urban settings worldwide (Prague Zoo 2016; Roshnath and Sinu 2017; Gentile 2022). They are listed as a special‐status species in 11 U.S. states and Ontario, Canada reflecting notable declines in the Great Lakes and Northeastern United States (IUCN 2019; Rahlin et al. 2022; Parsons 2023). Although typically considered crepuscular or nocturnal, BCNH increase their diurnal activity during the breeding season (Fasola 1984; Watmough 1978; Maccarone and Hamilton 2014). The leading threat to BCNH is habitat loss and degradation, as widespread conversion of wetland habitats for other land uses (i.e., agriculture, development) has significantly decreased the quantity and quality of potential BCNH breeding and foraging grounds (Parnell et al. 1988; Erwin 1995; Quinn et al. 2011). Due to their diverse diet, many BCNH populations now rely on human‐modified areas to obtain food resources (Watmough 1978; Bernick 2007; Taylor II et al. 2010; Maccarone and Hamilton 2014; Fidorra et al. 2016). As natural nesting habitats have been destroyed or abandoned, an increasing number of BCNH colonies have also been established in urban habitats (Kelly et al. 2006, Hunt 2016). BCNH colonies have been founded in cities across the globe, within parks, residential neighborhoods, commercial areas, harbors, and other urban waterways (Hunt 2016; Barber and Gross 2017; Curley 2013). Long‐term colonies have also been founded at zoos in multiple U.S. cities, Prague, Czech Republic, and Barcelona, Spain (Zoo Barcelona, n.d.; McConnell 2012; Hunt 2016; Prague Zoo 2016; Perkins and Mace 2017; Scarpignato et al. 2021).

Despite the unusual conditions under which some BCNH colonies occur, little information exists on local‐scale movements, space use, or activity patterns of individual BCNH in these anthropogenic landscapes. We used both location and accelerometer data collected from GPS/GSM satellite transmitters to investigate the behavior and ecology of BCNH in Chicago, Illinois to better understand the habitat use and activity patterns of this urban wading bird during the breeding season. Our study sought to: (a) quantify the home ranges of BCNH and assess the influence of body size, sex, and breeding status on home range size, (b) evaluate urban BCNH habitat use across a broad range of urban and semi‐natural environments, and (c) describe urban BCNH diel activity patterns during the reproductive period based on overall dynamic body acceleration (ODBA). We predicted that breeding status may influence home range size, as breeding birds may choose foraging grounds close to the colony to minimize energy expenditure during foraging trips (Piper 2011; Baert et al. 2021; Lalla et al. 2022; Yurek et al. 2024) and that breeding BCNH would show higher overall activity rates than non‐breeding birds due to the added demands of reproduction (Fasola 1984; Watmough 1978; Maccarone and Hamilton 2014). Understanding individual BCNH space use, habitat use, and activity while breeding in a high‐density urban landscape will provide important insights into their behavior and population dynamics that can inform conservation and management efforts of this declining species.

## 2 Materials and Methods

### 2.1 Study Site

All heron captures and processing took place at Lincoln Park Zoo (LPZ) in Chicago, IL, a city of 2.7 million residents (U.S. Census Bureau, n.d.). Since 2012, BCNH have nested in deciduous and coniferous trees within the Pritzker Family Children's Zoo above and proximate to the Red Wolf ( *Canis rufus* ) and American Black Bear ( *Ursus americanus* ) habitats (Slayton 2025). During the study period, colony size peaked at 686 (2023) and 695 (2024) adults and 486 (2023) and 485 (2024) chicks detected (Adams, personal communication, August 1, 2024). The LPZ colony is the largest remaining BCNH colony in the U.S. Great Lakes and the last major rookery in the state of Illinois.

### 2.2 Capture Method

BCNH were captured using 4′ × 4′ × 8′ walk‐in traps within the LPZ colony constructed from ¾” PVC piping and plastic poultry netting. Traps were baited daily, approximately two to three house before evening capture efforts, with 25‐30 frozen smelt (*Osmeridae spp.*) place in 8″ tall, 15 gal plastic stock tanks filled with 4‐6″ of water. Traps were constructed in the colony about two weeks before we attempted capture to habituate the birds to the structures and allow them to discover the bait. Trapping efforts took place from approximately 1700–2200 h or 0400–0900 h. During capture efforts, we monitored the traps remotely using a small camera or baby monitor while remaining concealed behind a large blind or in a nearby building. When a bird was completely inside the trap, a door built from 1” PVC and poultry netting was released via remote control and the bird was secured inside. Upon capture, researchers immediately began processing efforts. See Slayton (2025) for additional information on trap design and trapping methodology.

### 2.3 Heron Processing

Seventeen adult BCNH from the LPZ colony were captured, banded, and fitted with GPS/GSM trackers between the 2023 and 2024 BCNH breeding seasons. Each heron was fitted with standard USGS aluminum leg bands and a molded plastic band with unique alphanumeric codes, and the mass (kg) of each bird was recorded with a Pesola spring‐scale. Birds' tarsus length and culmen length were measured in millimeters using handheld calipers, and wing chord length was measured in millimeters using a large wing chord ruler. Four body feathers were also collected for sexing. Each bird was also equipped with a 16 g—18 g solar‐powered GPS/GSM transmitter manufactured by either Ornitela or Druid Technology. All transmitters were programmed to collect GPS locations every 60 min and upload data to a secure portal via cellular connection every 72 h. Transmitters were attached using “backpack” style Teflon harnesses and weighed less than 3% of each bird's body weight. This research was conducted in compliance with the University of Illinois Institutional Animal Care and Use Committee (Protocol #22044) and all necessary federal (U.S. Geological Survey Bird Banding Laboratory #23959) and state permits.

### 2.4 Breeding Status Determination

Reproductive timing within BCNH colonies is asynchronous, with birds initiating breeding over a one‐to‐two‐month period (Fasola 1984). At the LPZ colony, adults usually begin arriving in mid‐March and depart from the colony by the end of July (Adams, personal communication, August 1, 2024). Birds typically start laying eggs between the end of March and early May, with chicks fledging between early May and late July (Adams, personal communication, August 1, 2024). Since all tagged birds were captured in the colony, we presumed all were actively breeding, but further exploration of their GPS data suggested that six tagged birds did not appear to breed that year. Since direct behavioral observations were limited by colony density and logistical constraints, we inferred birds' breeding status based on their locations and movements after tagging (Koczur et al. 2018; Brzorad et al. 2015). Non‐breeding individuals were defined as those that left the colony within one week of tagging, rarely visited, and did not exhibit repeated visits to a consistent location (e.g., nest). This pattern indicated non‐breeding status due to prolonged absences incompatible with incubation or chick provisioning (Fasola 1984). Most fit this pattern, with one exception, see Slayton (2025). In contrast, breeding birds showed consistent movements between the colony and foraging sites for at least one week after tagging and spent long, continuous periods (> 20 h) in the colony. Although birds were tagged at different stages of the breeding cycle, it is unlikely that most non‐breeding birds could have completed a successful nesting attempt within the observed time frame, given the minimum fledging age of 29–34 days (Hothem et al. 2020; Slayton 2025).

### 2.5 Data Analyses

#### 2.5.1 Home Range Estimation and Habitat Classification

Birds' movements within a certain time frame (e.g., season, year) can be summarized and quantified as a home range, which is generally defined as the “area traversed by the individual in its normal activities of food gathering, mating, and caring for young” (Burt 1943). Three transmitters stopped uploading data within a few days of tagging, so home ranges were only calculated for the 14 birds (*n* = 8 breeding and *n* = 6 non‐breeding) that had more than seven days of consistent GPS data (*n* = 1 for 2023 and *n* = 13 for 2024). Breeding birds' home ranges were calculated based on GPS points collected between the date they were tagged until their final visit to the colony before departing from LPZ for the season. For non‐breeding birds, home ranges were estimated over the time interval starting from their final visit to the colony after tagging until their transmitter stopped uploading or 31 July 2024, when the BCNH breeding season ended and all adult BCNH had dispersed from the LPZ colony.

All data preparation and analyses were conducted in R version 4.4.2 (R Core Team 2024). Using continuous time movement modeling through the “ctmm” package in R (Calabrese et al. 2016), we produced autocorrelated kernel density estimations and 95% confidence intervals for both the 50% and 95% utilization distributions (UD) for each bird. The 50% UD represents the bird's “core” home range, where there is a 50% chance the bird will be in that area at any given time, while the 95% UD encompasses the bird's “total” home range (Calabrese et al. 2016). This method produces the estimated home range areas while accounting for potential data gaps due to transmitter failures or low battery, and for spatial and temporal autocorrelation between subsequent GPS points (Calabrese et al. 2016; Silva et al. 2022). Estimates for two non‐breeding birds that did not have a defined home range that they consistently used (effective sample size < 4) were calculated using parametric bootstrapping to improve estimates (Fleming et al. 2019).

#### 2.5.2 Sex Determination

Using the follicles of four scapular feathers plucked during bird processing, each bird's sex was determined using PCR to amplify the CHD‐1 gene, following methodologies outlined by Jensen et al. (2003). All lab work was carried out by the Avian Biotech division of Animal Genetics (Tallahassee, Florida, USA).

#### 2.5.3 Home Range Statistical Analysis

We used an Akaike's information criterion for small sample sizes (AICc) to assess the potential influence of bird body size based on tarsus length (Rising and Somers 1989), sex, and breeding status on the response variables of birds' estimated total and core home range sizes (Burnham and Anderson 2004). We constructed nine a priori candidate models based on hypothesized relationships between these variables and predicted that: (a) body size could influence home range size since larger individuals may need larger home ranges to find enough food to meet their higher energetic needs than smaller individuals (Harestad and Bunnel 1979; Gittleman and Harvey 1982; Ottaviani et al. 2006), (b) there could be differences in space use between sexes (Hothem et al. 2020), and (c) breeding and non‐breeding birds could show different space use patterns based on their use, or lack thereof, of the colony as a roost site (Stier et al. 2017). Additionally, we included a potential interaction between breeding status and sex to test whether there were different patterns within non‐breeding males and females since breeding BCNH share parental duties but are relatively solitary outside the reproductive period (Fasola 1984). We also included null and global models for comparison and used the same models to assess both total and core home range sizes. Both total and core home range estimates were log‐transformed to help normalize model residuals and stabilize their variance. We used the “nlme” package in R (Pinheiro and Bates 2025) to construct nine candidate generalized linear mixed models (GLMM) using individual bird ID as a random effect and used the “AICc” function from the package “MuMIn” (Bartoń 2025) to calculate AICc values for each model. Then, we calculated AICc weights (*w*_*i*_) for each model and identified the top models based on ∆AICc values less than two.

#### 2.5.4 Classification of GPS Points and Characterization of Birds' Habitat Use

Raw GPS data collected from the same 14 birds included in the home range analysis were uploaded and evaluated in ArcGIS Pro. Each GPS point's location was manually coded based on labels in Esri's Hybrid Reference Basemap or Google Earth satellite imagery. To accommodate for the uncertainty around each GPS point, all points collected within the boundaries of LPZ or directly across the street (within 100 m of the colony perimeter) were considered to be within the colony. All other data points were manually classified as either being within a city park or forest preserve, golf course, cemetery, industrial area, harbor or breakwater in Lake Michigan, location along the Lake Michigan shoreline, river or creek, school campus, residential street or complex, or commercial habitat type (see Appendix S1). This coded dataset was filtered to the same dates used for each bird's home range analysis, and all points collected within the colony were removed. The remaining GPS points were used to identify the specific locations used by each tagged bird outside the colony for foraging or roosting and to calculate their relative use of each habitat type. Relative use was calculated based on the proportion of each bird's total GPS points that were collected within each habitat type. Birds were classified as breeding or non‐breeding according to the same criteria used during the home range analysis. Additionally, the Haversine formula was used to compute the distance between each GPS point and the center of the colony.

#### 2.5.5 Diel Activity Patterns and ODBA

In addition to collecting GPS fixes, our GPS/GSM tags contained accelerometers that could be used to estimate animals' activity rates remotely, potentially capturing behaviors and activity changes that would be impossible to observe directly (Halsey et al. 2009; Schreven et al. 2021; Ozsanlav‐Harris et al. 2022). Only birds equipped with Druid Technology transmitters were included in activity analyses because the Ornitela transmitters failed to collect consistent accelerometer data within a few weeks of tagging. The Druid Technology tags measured birds' ODBA averaged over a 10‐min period. ODBA is calculated by summing an individual's dynamic acceleration across the X, Y, and Z axes (Jennings et al. 2021). Although ODBA values do not inherently contain information about specific behaviors, they can serve as a proxy for animal energy expenditure which provides insights into species' activity patterns and life history (Halsey et al. 2009; Scharf et al. 2016; Schreven et al. 2021; Ozsanlav‐Harris et al. 2022). When combined with GPS data, ODBA values can be used to infer more specific behaviors by integrating these measures of activity with a specific time and place (Scharf et al. 2016).

#### 2.5.6 Activity Data Preparation

ODBA data was downloaded from the 13 BCNH equipped with Druid Technology tags (*n* = 7 breeding and *n* = 6 non‐breeding) that collected more than seven days of consistent accelerometer readings. ODBA readings for breeding and non‐breeding birds were filtered to just include readings collected between the same dates used in their respective home range estimations. Each bird's ODBA values were assigned a time of day (TOD) using the “suncalc” package (Thieurmel and Elmarhraoui 2025). The earliest breeding birds' ODBA readings began on 15 May 2024 (ordinal day 136) and continued until the final tagged breeder left the colony on 20 July 2024 (ordinal day 202). Activity data for non‐breeding birds covered the time frame from when the first tagged bird dispersed from the colony on 24 May 2024 (ordinal day 145) until 31 July 2024 (ordinal day 213). Points collected between nautical dawn and sunrise were classified as “dawn”, between sunrise and sunset were classified as “day”, between sunset and nautical dusk were classified as “dusk”, and points between nautical dusk and nautical dawn were classified as “night.” Once all individual ODBA values were classified, they were averaged over each TOD for every day between the date each bird was tagged until their transmitter stopped uploading new data, or until 31 July 2024 (i.e., average ODBA for dawn, day, dusk, and night for every date). We used the average ODBA as a proxy for overall energy expenditure during each TOD to more clearly compare activity levels between individuals and over time, since the raw ODBA values only reflected activity for 10‐min intervals.

#### 2.5.7 Activity Statistical Analysis

We used an information‐theoretic approach to consider the effects of breeding status, TOD, and ordinal day on the response variable of average ODBA to investigate how tagged BCNH diel activity patterns differed between individuals and over time (Burnham and Anderson 2004). We included these variables in 12 a priori candidate models because we predicted that (a) birds' activity levels would be higher during dawn, dusk, and night than during the day because BCNH are often crepuscular or nocturnal, (b) activity levels would differ between breeding and non‐breeding birds since reproductive activities require high amounts of additional energy expenditure, and (c) birds' activity would change as the reproductive period progressed and breeding activities intensified. We also included several interaction models between all three of these variables to account for potential differences in activity at different times of day or over the reproductive period while birds were breeding vs. not breeding. Averaged ODBA values for each TOD were log‐transformed to help normalize model residuals and stabilize their variance. For each candidate model, we constructed GLMMs using the “nlme” package (Pinheiro and Bates 2025) and included individual bird ID as a random effect. Then we used the “AICc” function from the package “MuMIn” (Bartoń 2025) to calculate AICc values for each model, calculated their AICc weights (*w*_*i*_) for each model, and identified the top models based on ∆AICc values less than two.

## 3 Results

**FIGURE 1:**
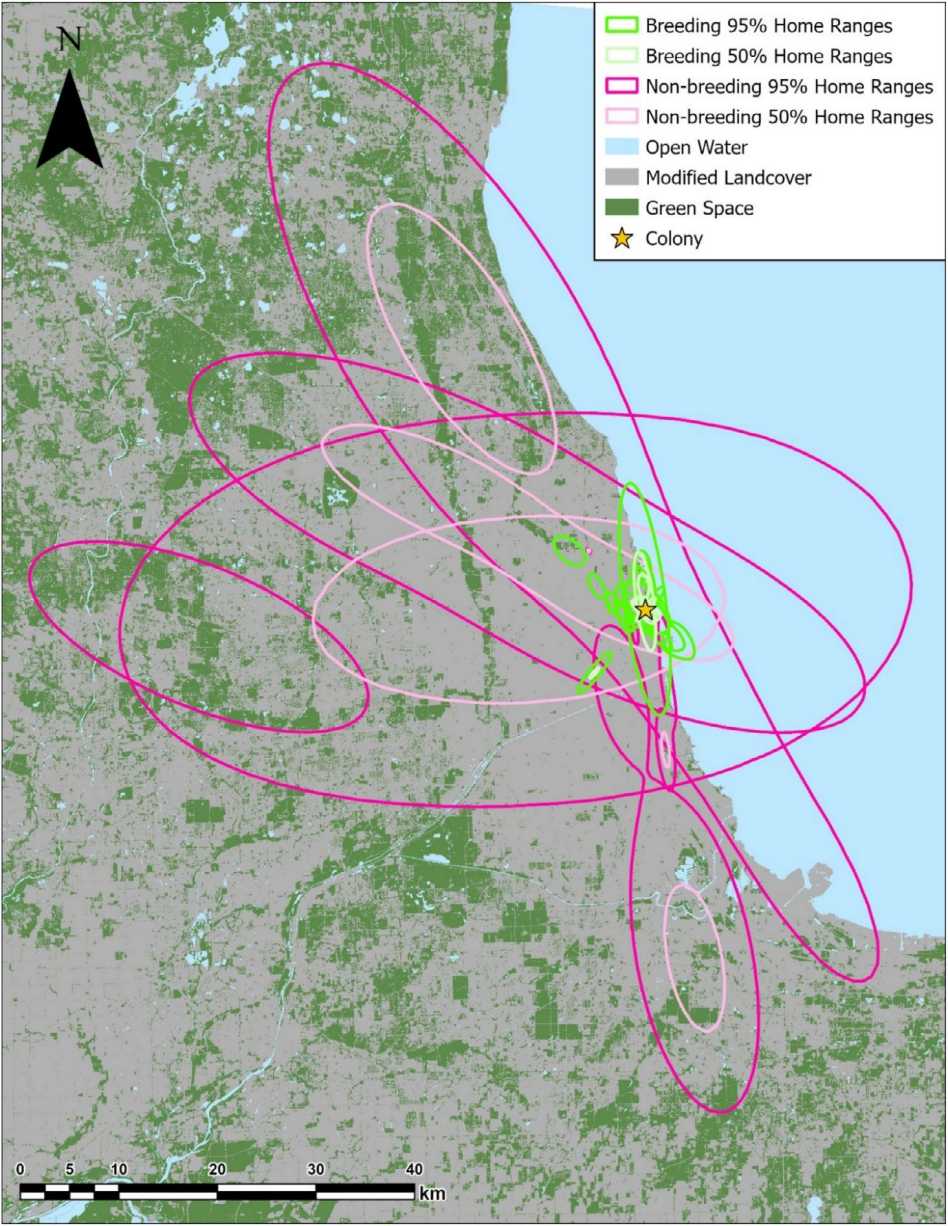
Map of 14 black‐crowned night herons ( *Nycticorax nycticorax* ; BCNH) total [95% UD] and core [50% UD] estimated home ranges calculated from GPS data collected from seven breeding BCNH (green, lime) and six non‐breeding BCNH (magenta, light pink) in Chicago, Illinois in 2023 (*n* = 1) and 2024 (*n* = 13). Background raster data was downloaded from the Multi‐Resolution Land Characteristics Consortium Raster (30 m × 30 m, Albers Equal Area Projection, North American Datum 1983) and reclassified as modified (gray) or naturalized (green) landcover.

We estimated home ranges for eight breeding (*n* = 5 male and *n* = 3 female) and six non‐breeding (*n* = 4 male and *n* = 2 female) birds. BCNH home ranges estimates were highly variable (Table 1; Appendix S1). While there was large individual variation, the best supported model indicated that variation in both total and core home range size was closely associated with breeding status (total [95% UD]: *w*_*i*_ = 0.67, marginal *R* ^2^ = 0.46; core [50% UD]: *w*_*i*_ = 0.67, marginal *R* ^2^ = 0.44; Tables 2, 3). The estimated home range sizes for non‐breeders' total home ranges were about 18 times larger than breeders', and non‐breeders' core home ranges were about 25 times larger than breeders' (Figure 1). Neither tarsus length (mean = 81.7 mm, SD = 4.44 mm, range = 73.8–89.7 mm) nor sex were important predictors of either home range size. The combination of breeding status and the random effect of individual bird ID explain almost all the variation in birds' home range size (conditional *R* ^2^ = 0.93).

**TABLE 1:** Summary of estimated total [95% UD] and core [50% UD] home range sizes of 14 black‐crowned night herons ( *Nycticorax nycticorax* ) tagged at the LPZ rookery in 2023–2024.

Bird status	#Days	#Fixes	Total home range (km^2^)			Core home range (km^2^)		
	Mean	Mean	Mean	SD	Range	Mean	SD	Range
Breeding	26	475	23.98	23.64	1.9–78.51	3.80	4.35	0.28–14.21
Non‐breeding	54	765	1047.95	1062.45	9.79–2559.65	220.48	245.73	1.79–623.48

*Note:* Home range estimates were calculated using the continuous time movement modeling package “ctmm” in R.

**TABLE 2:** AICc results from the generalized linear mixed model analysis of tagged black‐crowned night herons' ( *Nycticorax nycticorax* ) estimated total [95% UD] home ranges based on GPS data collected in 2023 (*n* = 1) and (*n* = 13).

Model name	K	AICc	ΔAICc	*w*_*i*_
Breeding Status	2	65.61	0	0.67
Breeding Status * Sex	4	69.38	3.78	0.10
Null	1	69.73	4.13	0.09
Breeding Status + Sex	3	70.50	4.89	0.06
Breeding Status + Tarsus	3	70.64	5.04	0.05
Tarsus	2	73.65	8.05	0.01
Sex	2	73.74	8.13	0.01
Breeding Status + Tarsus + Sex	4	77.00	11.39	0.00
Sex + Tarsus	3	78.70	13.10	0.00

*Note:* All models included a random effect of individual bird ID. Asterisk indicates an interaction effect between the two predictor variables.

**TABLE 3:** AICc results from the generalized linear mixed model analysis of tagged black‐crowned night herons' ( *Nycticorax nycticorax* ) estimated core [50% UD] home ranges based on GPS data collected in 2023 (*n* = 1) and (*n* = 13).

Model name	K	AICc	ΔAICc	*w*_*i*_
Breeding Status	2	67.91	0	0.67
Null	1	71.54	3.63	0.11
Breeding Status + Sex	3	72.55	4.65	0.07
Breeding Status * Sex	4	72.60	4.70	0.06
Breeding Status + Tarsus	3	72.90	4.99	0.06
Tarsus	2	75.39	7.49	0.02
Sex	2	75.44	7.53	0.02
Breeding Status + Tarsus + Sex	4	79.05	11.15	0.00
Sex + Tarsus	3	80.39	12.48	0.00

*Note:* All models included a random effect of individual bird ID. Asterisk indicates an interaction effect between the two predictor variables.

**FIGURE 2:**
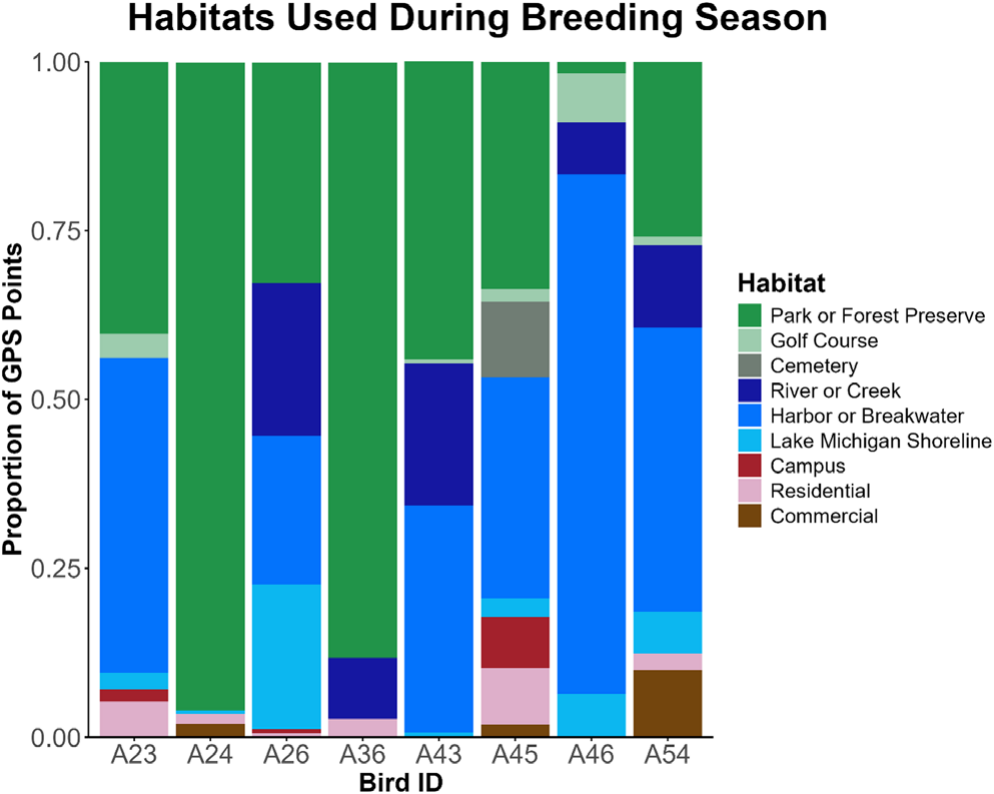
Proportion of habitat types used by eight breeding black‐crowned night herons ( *Nycticorax nycticorax* ) based on GPS points collected by GPS/GSM transmitters in 2023 (*n* = 1) and 2024 (*n* = 13). GPS points were divided by the total number of points collected by each transmitter while they were breeding or until the transmitter failed. Bird ID refers to each individual's alphanumeric band code.

Over the course of the breeding season, tagged BCNH used a variety of habitat types across Chicago and neighboring areas when they were not in the colony. Urban green spaces such as city parks, forest preserves, golf courses, and cemeteries made up the highest proportion of breeding birds' habitat use (mean = 54.2% of GPS points, SD = 26.9%, range = 27.1%–95%), followed by harbors, breakwaters, or beaches on Lake Michigan (mean = 33.2% of GPS points, SD = 25.5%, range = 0%–70.7%), and sections of the Chicago River (mean = 9.2% of GPS points, SD = 9.9%, range = 0%–22.6%; Figure 2). Breeding birds used habitats that were an average distance of 3.62 km from the colony (SD = 2.12 km, range = 0.84–8.03 km), likely choosing foraging grounds closer to the colony to maximize foraging efficiency.

**FIGURE 3:**
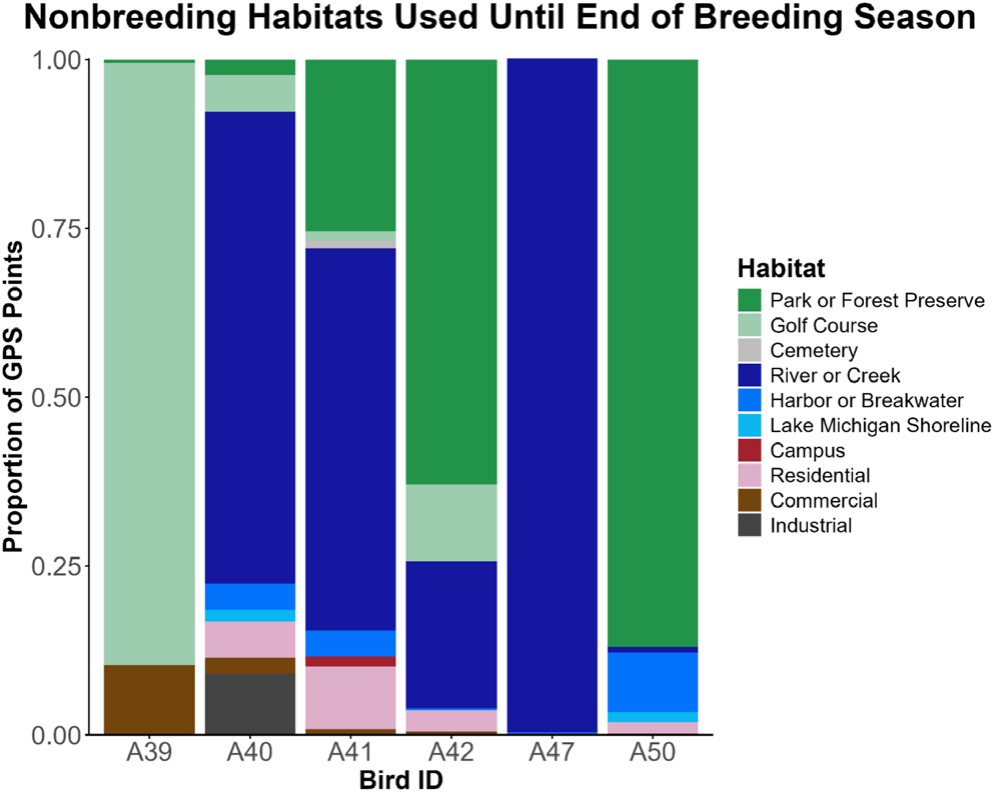
Proportion of habitat types used by six non‐breeding black‐crowned night herons ( *Nycticorax nycticorax* ) based on GPS points collected by GPS/GSM transmitters in 2023 (*n* = 1) and 2024 (*n* = 13). GPS points were divided by the total number of points collected by each transmitter while they were breeding or until the transmitter failed. Bird ID refers to each individual's alphanumeric band code.

In general, non‐breeding birds used many of the same types of habitats as breeding birds (Figure 3), but they also used areas that, on average, were farther from the colony (Wilcoxon‐signed rank test, W = 0, *n* = 14, *p* < 0.001). Habitats used by non‐breeding birds were an average of 21.31 km from the colony (SD = 12.10 km, range = 8.28–35.14 km), suggesting that they chose foraging habitats farther away once they were not restricted in their foraging range by proximity to the colony. Non‐breeding birds also frequently used urban green spaces (mean = 48.7% of total GPS points, SD = 39.6%, range = 0%–89.5%) and rivers or creeks (mean = 41.5%, SD = 40.5%, range = 0%–99.8%) for foraging and roosting. However, non‐breeding birds generally spent less time foraging in habitats along Lake Michigan (mean = 3.4% of total GPS points, SD = 4.1%, range = 0%–10.3%).

**FIGURE 4:**
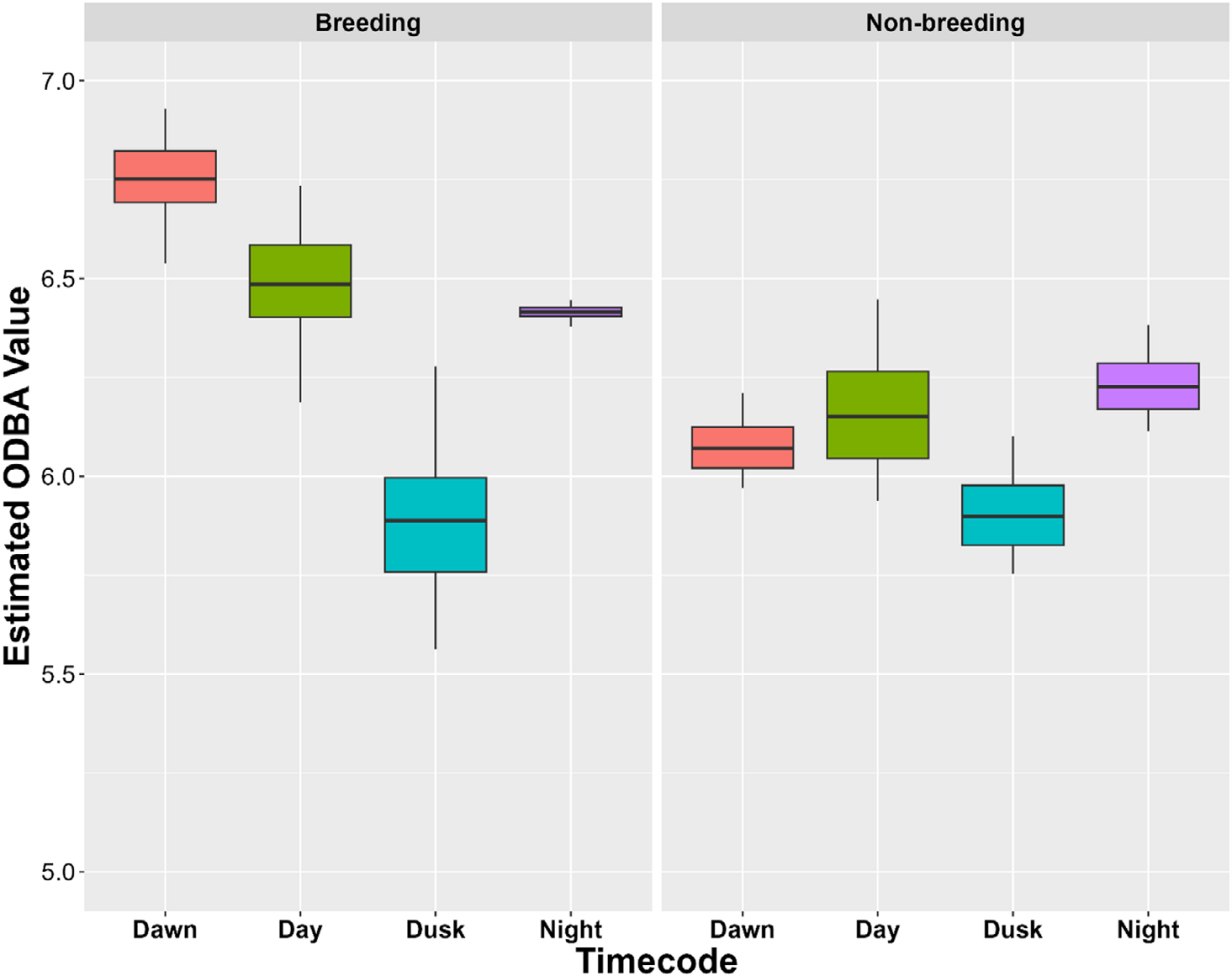
Estimated mean overall dynamic body acceleration (ODBA) values while tagged black‐crowned night herons ( *Nycticorax nycticorax* ) were breeding and not breeding, based on accelerometer data collected in 2024. Estimated ODBA is shown on a logarithmic scale.

Accelerometer data from 13 individuals tagged in 2024 revealed clear variation in ODBA across the 24‐h cycle. Our best supported model suggests that variation in birds' average ODBA was influenced by interactions between TOD, breeding status, and ordinal date (*w*_*i*_ = 1.0, marginal *R* ^2^ = 0.21, conditional *R* ^2^ = 0.28, Table 4). Using this model, we estimated mean values of logODBA at dawn, day, dusk, and night during the breeding and non‐breeding periods, and over the course of the breeding (ordinal days 136–202) or non‐breeding (ordinal days 145–213) time frames. Breeding BCNH had higher estimated ODBA values during dawn (*p* < 0.0001, SE = 0.10) and daytime (*p* < 0.01, SE = 0.10) than non‐breeding BCNH (Figure 4). There was no difference in average activity at dusk (*p* = 0.29, SE = 0.10) or nighttime (*p* = 0.11, SE = 0.10) between breeding statuses.

**TABLE 4:** AICc results from the generalized linear mixed model analysis of tagged black‐crowned night herons' ( *Nycticorax nycticorax* ) diel activity analysis based on overall dynamic body acceleration data collected in 2024.

Model	K	AICc	ΔAICc	*w*_*i*_
Time of Day*Julian Date*Breeding Status	8	2868.79	0	1
Breeding Status*Time of Day	4	2926.56	57.77	0
Time of Day + Breeding Status + Julian Date	4	2995.79	127.00	0
Julian Date + Time of Day	3	2999.32	130.53	0
Julian Date*Time of Day	4	3003.50	134.71	0
Breeding Status + Time of Day	3	3025.60	156.81	0
Time of Day	2	3031.68	162.90	0
Breeding Status*Julian Date	4	3174.73	305.94	0
Breeding Status + Julian Date	3	3180.35	311.60	0
Julian Date	2	3184.03	315.24	0
Breeding Status	2	3206.91	338.12	0
Null	1	3213.10	344.31	0

*Note:* All models included a random effect of individual bird ID. Asterisk indicates an interaction effect between the two predictor variables.

While birds were breeding, they had the highest average ODBA at dawn relative to other times of day (day: *p* < 0.01, SE = 0.06; dusk: *p* < 0.001, SE = 0.06; night: *p* < 0.0001, SE = 0.06). BCNH activity at night was higher than dusk (*p* < 0.0001, SE = 0.06), but not statistically different from their activity during the day (*p* = 0.18, SE = 0.06). Birds' average ODBA during dawn (*p* = 0.32, SE < 0.08) and the night (*p* = 0.99, SE < 0.08) did not change over the course of the breeding season (Appendix S1). There was a non‐significant trend for increased activity during the day (*p* = 0.06, SE < 0.08), while bird activity at dusk decreased over the course of the breeding season (*p* < 0.01, SE < 0.08).

Non‐breeding birds did not exhibit the same degree of variation in activity levels throughout the 24‐h period as breeding birds (Appendix S1). Similar to the breeding period, there was no difference between activity during daytime hours and nighttime hours (*p* = 0.42, SE = 0.04), but birds showed slightly higher average ODBA during nighttime than at dawn (*p* < 0.01, SE = 0.04) and dusk (*p* < 0.001, SE = 0.04). Birds were still more active during the day (*p* < 0.001, SE = 0.04) and dawn (*p* = 0.001, SE = 0.04) than at dusk. Birds' average ODBA during the day and at dusk decreased over the course of the non‐breeding period (day: *p* < 0.001, SE < 0.05; dusk: *p* = 0.02, SE = 0.03; Appendix S1). The average ODBA at dawn and night during the non‐breeding period declined, but the trends were not significant (dawn: *p* = 0.13, SE < 0.08; night: *p* = 0.06, SE < 0.05).

## 4 Discussion

Urban landscapes present a unique set of challenges for breeding BCNH, yet little is known about how this wetland species successfully exploits cities during the breeding season. This study provides new insights into the movement ecology, habitat use, and diel activity patterns of BCNH breeding within a highly urbanized landscape. Breeding BCNH maintained relatively compact home ranges centered on the colony and adjacent water bodies, while non‐breeding individuals ranged widely and used habitats further from the colony. While both groups were active throughout the 24‐h period, breeding birds displayed relatively higher activity at dawn, during the day, and at night, while non‐breeders exhibited a more evenly distributed pattern of activity. Tagged BCNH also showed a high degree of individual variation in their movements and habitat use during the reproductive period, highlighting this species' behavioral flexibility that likely contributes to their ability to adapt to and establish colonies in urban areas around the world.

Previous studies have found that birds' movements and their resulting home ranges can vary based on a range of physiological or environmental factors such as age, sex, body size, genetics, breeding status, resource availability, landscape composition, and population density (Mazerolle and Hobson 2004; Baert et al. 2021; Mirski et al. 2021; Peng et al. 2023). In this study, tagged birds' home ranges during the reproductive period varied from two km^2^ areas near the colony to thousands of km^2^ spanning wide swaths of urban area. Large variation in home range size has also been observed in wintering BCNH and other heron species. Studies of Agami Herons ( *Agamia agami* ) and Gray Herons ( *Ardea cinerea* ) during the breeding season found their home ranges could range from 100 to 200 km^2^ (*n* = 3) and 3.7–755.7 km^2^ (*n* = 4) respectively (Stier et al. 2017; Lim et al. 2021). Stein (2018) also found high levels of variation in the wintering home range size of BCNH, estimating their total home ranges to be between 4.42–1527.63 km^2^ and their core home ranges between 0.93–381.9 km^2^ (*n* = 13). However, none of these studies explored potential drivers of this variation during the breeding season.

Here, we assessed the potential influence of body size, sex, and breeding status on this variation in BCNH home range size. Although tagged birds' tarsus lengths ranged between 73.8–89.7 mm, this variable was not a good predictor of home range size. While some general home range studies have suggested a positive relationship between body size and home range size (Harestad and Bunnel 1979; Ottaviani et al. 2006), other tracking studies of waterbirds have not found this relationship (Mander et al. 2022; Peng et al. 2023). Sex was also not a strong predictor of home range size. Since BCNH pairs share parental care duties, both sexes likely demonstrate similar reproductive and foraging behaviors and therefore may not differ as much in terms of the area of their overall space use (Fasola 1984; Hothem et al. 2020).

However, we observed clear behavioral differentiation between breeding and non‐breeding individuals. This contrast likely reflects the central‐place foraging constraints imposed by nest attendance and chick provisioning (Orians and Pearson 1979; Lalla et al. 2022). Breeding BCNH in this study also traveled relatively short distances from the colony to foraging habitats that were on average 3.62 km away, with a maximum of 10 km away from LPZ. Breeding BCNHs' relatively high foraging site fidelity and use of habitats close to the colony likely contribute to their smaller home range sizes (Piper 2011, Lalla et al. 2022).

Conversely, non‐breeding birds were not limited in their foraging range by proximity to a central location after leaving the colony and showed greater mobility and exploratory movements as they searched for alternative foraging or roosting habitats or prospected for alternative nesting sites (Reed et al. 1999). This may explain why some non‐breeding birds had much larger core and total home ranges than breeding BCNH after they departed from the colony. Tracking studies of other colonial waterbirds have observed comparable differences between breeding and non‐breeding individuals. Baert et al. (2021) found that foraging behavior of Lesser Black‐backed Gulls ( *Larus fuscus* ) diverged between breeders and non‐breeders, where breeding birds showed higher site fidelity to a smaller number of foraging grounds than non‐breeders, who demonstrated more exploratory, generalist foraging behaviors. Non‐breeding European White Stork (Zurell et al. 2018) and Brown Skua ( *Stercorarius antarcticus* ; Lamb et al. 2023) also had larger home ranges than breeders of both species.

While these home ranges estimate general space use by BCNH in Chicago, it is also important to examine the specific habitats birds used during the reproductive period. The broad habitat use we observed is consistent with prior research indicating that BCNH readily exploit diverse foraging environments (Fidorra et al. 2016; Rahlin et al. 2022). All breeding birds spent at least some of their time outside the colony in urban green spaces that contain relatively shallow ponds, lagoons, or other urban wetlands that offer predictable foraging opportunities for birds. These constructed waterbodies often provide ideal foraging conditions for BCNH: shallow water with emergent vegetation, manicured shorelines that make hunting easier, and natural or artificial perches into the water that birds can use to hunt (White and Main 2005; Dwyer et al. 2013; Maccarone and Hamilton 2014; Herteux et al. 2020). Only 9.5% of the total area within 10 km of the colony consists of green space containing or adjacent to water, so these areas concentrate foraging breeding birds and serve as critical habitats for the species in Chicago.

Additionally, many breeding birds foraged in larger urban water bodies, including Lake Michigan and sections of the Chicago River, which are known to contain crawfish and other relevant fish prey (Happel et al. 2024). Chicago's harbors along Lake Michigan are also common spawning grounds for a variety of fish species, such as alewives ( *Alosa pseudoharengus* ), leading to opportunistic feasting opportunities for breeding birds (Savitz et al. 1996). Although most sections of Lake Michigan and developed portions of the Chicago River lack shallow shorelines, various anthropogenic structures (e.g., boat docks, concrete sidewalks, city breakwaters) serve as artificial perches from which birds may hunt, allowing them to forage in otherwise inaccessible bodies of water (Kushlan 1973; Maccarone and Hamilton 2014). Additionally, anthropogenic lighting at the edges of these waterbodies likely attract fish, concentrating them towards the shoreline and making them more accessible to birds (Fidorra et al. 2016; Erwin et al. 1991; Dwyer et al. 2013).

Non‐breeding birds used similar habitat types to breeding birds but mostly chose areas farther from the colony. Each non‐breeding BCNH used urban green spaces after leaving the colony, with several birds using golf courses at higher proportions than breeders. Non‐breeders also used sections of rivers or creeks but were generally less concentrated along the Chicago River and rarely used habitats along Lake Michigan. This habitat use is consistent with the fact that non‐breeding birds chose habitats that were farther away from the most highly developed part of the city once they were no longer tied to the colony as a central roost site. Non‐breeders may have traveled to more distant foraging grounds to reduce direct competition over resources with breeding birds closer to the colony (Burger 1981), and to take advantage of the higher density of wetland or riverine areas that also provide significant tree cover for roosting (Endo et al. 2006). Although these habitats were further away from the most densely populated portions of Chicago, non‐breeding birds continued to use relatively urban and suburban habitats surrounding the city rather than move to more natural wetland habitats in the region.

In addition to exploring BCNH home range size and habitat use, we also examined BCNH diel activity patterns during the reproductive period. BCNH are typically considered to be crepuscular or nocturnal, with their highest levels of activity occurring between dusk and dawn and resting during the daytime (Watmough 1978; Rojas et al. 1999; Maccarone and Hamilton 2014; Hothem et al. 2020). This has been proposed as an adaptation to reduce foraging competition with diurnal waders, with whom they often nest and share foraging grounds (Fasola 1984; Watmough 1978; Samraoui et al. 2012). Despite the assumption that “night herons” are most active at night, our ODBA analysis suggests that BCNH in this study were moving around throughout the 24‐h period, regardless of breeding status. However, they do show changes in their average activity between different times of day and over the course of the reproductive period.

We found that birds' average activity and energy expenditure at dawn and during the daytime were significantly higher while birds were engaging in breeding activities. Notably, the highest average ODBA readings occurred at dawn during the breeding season. This could be because peak feeding activity takes place at dawn, as was reported in a study of a BCNH colony in Italy (Fasola 1984). Additionally, many birds departed for or returned from the foraging grounds around dawn, and those flights would have caused peaks in ODBA. Other studies have shown that breeding BCNH spend more time foraging during the daytime than at other times of year to meet higher energy needs of themselves and chicks (Fasola 1984; Watmough 1978; Endo et al. 2006; Maccarone and Hamilton 2014). The higher rates of around‐the‐clock foraging activities likely played a role in birds' overall higher average ODBA during the day and at dawn, but we also found that many of the highest raw ODBA readings from breeding birds were collected while they were in the colony during these times. These bursts in activity could be caused by other reproductive and chick‐rearing behaviors in the colony, when birds may be flying up and down from the ground to collect sticks for their nests, building their nests, feeding young, or engaging with conspecifics. All of these behaviors could explain higher daytime activity rates in breeding birds than non‐breeding birds.

Non‐breeding birds also showed similar levels of activity during the day and at night. This is surprising, given that non‐breeding birds would be expected to revert to a more nocturnal circadian rhythm once they no longer had to engage in reproductive activities. Although BCNH are known for their nocturnal habits and good night vision, there is no physiological limitation stopping them from being active during the day. BCNH have larger, wider‐set eyes than other ardeids and several vision genes that have adapted to improve vision under dim light conditions, but their vision is similar to that of diurnal herons during the day (Katzir and Martin 1998; Luo et al. 2022). Therefore, it is possible that this species is uniquely able to adapt their diel activity patterns when living in anthropogenic habitats (Endo et al. 2006; Dorn et al. 2011; Fuirst et al. 2018; Marín‐Gómez et al. 2020). The Chicago BCNH may also face less competition with diurnal waders within the city than other populations throughout the species' breeding range, which may allow them to take advantage of more foraging opportunities during the day. Though Chicago BCNH share these foraging grounds with urban Great Blue Herons ( *Ardea herodias* ) and Green Herons ( *Butorides virescens* ), these species likely go after larger or smaller prey items than BCNH. Therefore, these BCNH may face less direct competition with other similarly sized species that they nest and forage with in more southern parts of their North American range (Harmon et al. 2022).

Throughout this study, we attempted to identify and characterize patterns in BCNH home range size, habitat use, and activity that could be generalized and applied to birds breeding in a highly urbanized environment. However, these birds also demonstrated a high degree of individual variation in their behavior and decision‐making. These differences may be driven by a wide range of factors we could not measure, including age, genetics, health status, or personality (Ryder 1980; Shaw 2020; Baert et al. 2021). One of the clearest examples of this was in the decision of tagged BCNH to breed or not breed. Adults that do not breed in a given year, also called “floaters,” are very common within seabird colonies (Aebischer and Wanless 1992; Reed et al. 1999; Baert et al. 2021; Lamb et al. 2023; Ainley et al. 2024), and non‐breeding adults were reported in another urban BCNH colony in New York City (Bernick 2007). Individuals may not breed due to poor health, high competition for limited nesting habitat, failure to find a mate, late return from migration, or after suffering early nest failure (Reed et al. 1999; Ainley et al. 2024). Early colony departure could also relate to birds' age or experience, where younger or more inexperienced birds may be more likely to allocate more energy towards breeding later in life (Curio 1983; Clutton‐Brock 1984; Weimerskirch 1992). Given their relatively long lifespan (average 3–15 years; Hothem et al. 2020), migratory nature, high competition for nest sites within the colony, and no other established colonies nearby, any of these factors could have caused some birds not to breed in 2024.

Although urban areas pose numerous challenges for wildlife, the BCNH's ability to exploit urban landscapes for both nesting and foraging, combined with their flexibility in habitat use and tolerance of human activity, has allowed them to maintain robust breeding populations in urban centers (McKinney 2002; Evans et al. 2011; Ducatez et al. 2018; Callaghan et al. 2019; Patankar et al. 2021). Such habitat flexibility likely contributes to the persistence of the LPZ colony, which has continued to grow for more than a decade despite intensive public disturbance and limited natural habitat. The small home ranges of breeding birds and their reliance on multiple nearby foraging sites suggest that, despite being highly modified, these urban habitats can provide consistent and accessible food and reproductive resources with relatively low predation risk (Kelly et al. 2006; Hernandez et al. 2016). Moreover, the continued use of developed areas by non‐breeding individuals after dispersing from the colony reinforces the high value of resource‐rich urban habitats for this population.

Although the Chicago population appears stable (Hunt 2016, Adams, personal communication, August 1, 2025), our results underscore the importance of targeted management efforts to support the continued persistence of BCNH in Chicago and other major urban areas. These habitats may expose birds to higher risks of anthropogenic threats such as commercial and industrial pollutants in waterways or remnants of fishing hooks and line that can trap or injure foraging birds. Care should be taken to reduce the influx of aquatic environmental pollutants and improve enforcement of safe fishing practices. This study highlights the diverse behavioral strategies that likely contribute to the success of BCNH in urban areas, including flexible diel activity patterns and the ability to thrive in a variety of anthropogenic habitat types.

## Author Contributions

**Sarah Slayton:** conceptualization (equal), formal analysis (lead), project administration (equal), writing – original draft (lead). **Henry Adams:** conceptualization (equal), project administration (equal), writing – review and editing (supporting). **Michael Avara:** conceptualization (equal), project administration (supporting), writing – review and editing (supporting). **Brad Semel:** conceptualization (equal), project administration (supporting), writing – review and editing (supporting). **Amy Lardner:** funding acquisition (supporting), project administration (supporting), writing – review and editing (supporting). **Liza Lehrer:** funding acquisition (supporting), project administration (supporting), writing – review and editing (supporting). **Michael Ward:** conceptualization (equal), formal analysis (supporting), funding acquisition (supporting), project administration (supporting), supervision (lead), writing – review and editing (lead).

## Funding

This work was supported by the Bird Conservation Network, Lincoln Park Zoo, Illinois Department of Natural Resources, College of Agricultural, Consumer and Environmental Sciences, University of Illinois at Urbana‐Champaign, Illinois Audubon Society.

## Conflicts of Interest

The authors declare no conflicts of interest.

## Supporting information


**Appendix S1:** ece373310‐sup‐0001‐AppendixS1.docx.

## Data Availability

Data used to complete the home range, habitat use, and activity analyses are available through Illinois Data Bank at https://doi.org/10.13012/B2IDB‐9027147_V1. GPS data can be accessed through the Movebank Data Repository at https://doi.org/10.5441/001/1.737. Code for these analyses can be found in the Supporting Information.
