# Supplementary material for: The Windy City Rookery: Movement and Activity Patterns of Black‐Crowned Night Herons (Nycticorax nycticorax) in a Human‐Dominated Landscape
Source: Ecol Evol. 2026 Mar 31;16(4):e73310. doi: 10.1002/ece3.73310 (PMC13106992; doi:10.1002/ece3.73310)
Supplement: Supplementary file 1 — Appendix S1: ece373310‐sup‐0001‐AppendixS1.docx. [file ECE3-16-e73310-s001.docx]

**Appendix**

Table 1**:** Descriptions of habitat types used to characterize tagged black-crowned night herons’ (*Nycticorax nycticorax*) GPS locations.

| **Habitat Type** | **Description** |
| --- | --- |
| Park or Forest Preserve | Tracts of publicly owned (e.g. Chicago Park District, Cook County Forest Preserve) land or natural areas that are designated for recreation and/or habitat protection. Many contain natural features such as ponds, wetlands, and/or forest fragments. |
| Golf Course | Private or publicly owned green space used to play golf. Typically contain shallow ponds or wetland areas that may serve as obstacles to game play and/or provide wildlife habitat. |
| Cemetery | Private or publicly owned green space used for burials. Often contain shallow ponds or wetland areas used by visitors as areas for reflection. |
| River or Creek | Narrow body of water that flows across the landscape. May refer to smaller, natural or restored stretches of water or channelized sections that flow through the study area. |
| Harbor or Breakwater | Anthropogenic structures or features near the Lake Michigan shoreline to store boats or prevent wave action. |
| Lake Michigan Shoreline | Any area along the perimeter of Lake Michigan that was not a harbor. Could include beaches or piers that provide direct access to the water. |
| Campus | Area of land that was designated as a school. Could include elementary schools up to college campuses. |
| Residential | Any location that could not be attributed to any of the categories described above but was surrounded by houses or apartment buildings rather than commercial development (e.g. street trees, subdivisions) |
| Commercial | Any location that could not be attributed to any of the categories described above but was surrounded by commercial development such as storefronts or offices. |
| Industrial | Areas that are primarily characterized by heavy industry and manufacturing (e.g. ArcelorMittal steel facility) |

Table 2: Estimated total [95% UD] and core [50% UD] home range sizes of 14 black-crowned night herons (*Nycticorax nycticorax*) tagged at the LPZ rookery in 2023 (n=1) and 2024 (n=13). Home range estimates and their respective 95% confidence intervals were calculated using the continuous time movement modeling package “ctmm” in R.

| **Bird ID** | **Sex** | **Tarsus Length (mm)** | **Breeding?** | **Estimated 95% Home Range (km^2^)** | **95% UD: Lower 95% CI** | **95% UD: Upper 95% CI** | **Estimated 50% Home Range (km^2^)** | **50% UD: Lower 95% CI** | **50% UD: Upper 95% CI** |
| --- | --- | --- | --- | --- | --- | --- | --- | --- | --- |
| A39 | M | 86 | N | 362.90 | 75.29 | 872.32 | 64.88 | 13.46 | 155.97 |
| A40 | F | 83 | N | 1670.00 | 576.45 | 3334.73 | 285.79 | 98.65 | 570.69 |
| A41 | M | 81 | N | 1661.34 | 1110.01 | 2322.06 | 344.91 | 230.44 | 482.08 |
| A42 | F | 75.7 | N | 2559.65 | 92.14 | 8977.10 | 623.48 | 22.44 | 2186.64 |
| A47 | M | 81.2 | N | 9.79 | 8.75 | 10.88 | 1.79 | 1.60 | 1.98 |
| A50 | M | 80.3 | N | 23.98 | 19.24 | 29.23 | 2.00 | 1.60 | 2.43 |
| A23 | M | 84.9 | Y | 78.51 | 40.67 | 128.58 | 14.21 | 7.36 | 23.27 |
| A24 | F | 74.9 | Y | 9.46 | 8.10 | 10.90 | 1.78 | 1.53 | 2.06 |
| A26 | M | 84.9 | Y | 17.84 | 10.73 | 26.73 | 3.60 | 2.16 | 5.39 |
| A36 | F | 83.5 | Y | 1.90 | 1.40 | 2.47 | 0.28 | 0.30 | 0.37 |
| A43 | M | 89.7 | Y | 15.57 | 11.91 | 19.70 | 1.51 | 1.15 | 1.91 |
| A45 | M | 73.8 | Y | 24.20 | 19.08 | 30.38 | 3.03 | 2.37 | 3.77 |
| A46 | F | 82.1 | Y | 14.29 | 9.38 | 20.21 | 2.71 | 1.80 | 3.83 |
| A54 | M | 82.8 | Y | 30.04 | 21.65 | 39.79 | 3.28 | 2.36 | 4.34 |

Table 3: Summary of overall dynamic body acceleration (ODBA) data collected from 13 Druid Technology GPS/GSM transmitters deployed on black-crowned night herons (*Nycticorax nycticorax)* in 2024.

| **Status** | **# Days ODBA Collection** | | | **# ODBA Readings** | | |
| --- | --- | --- | --- | --- | --- | --- |
|  | Mean | SD | Range | Mean | SD | Range |
| Breeding | 22 | 13 | 7 – 44 | 3,028 | 1,726 | 968 – 5,717 |
| Non-breeding | 56 | 12 | 40 – 68 | 7,802 | 1,819 | 5,163 – 9,705 |


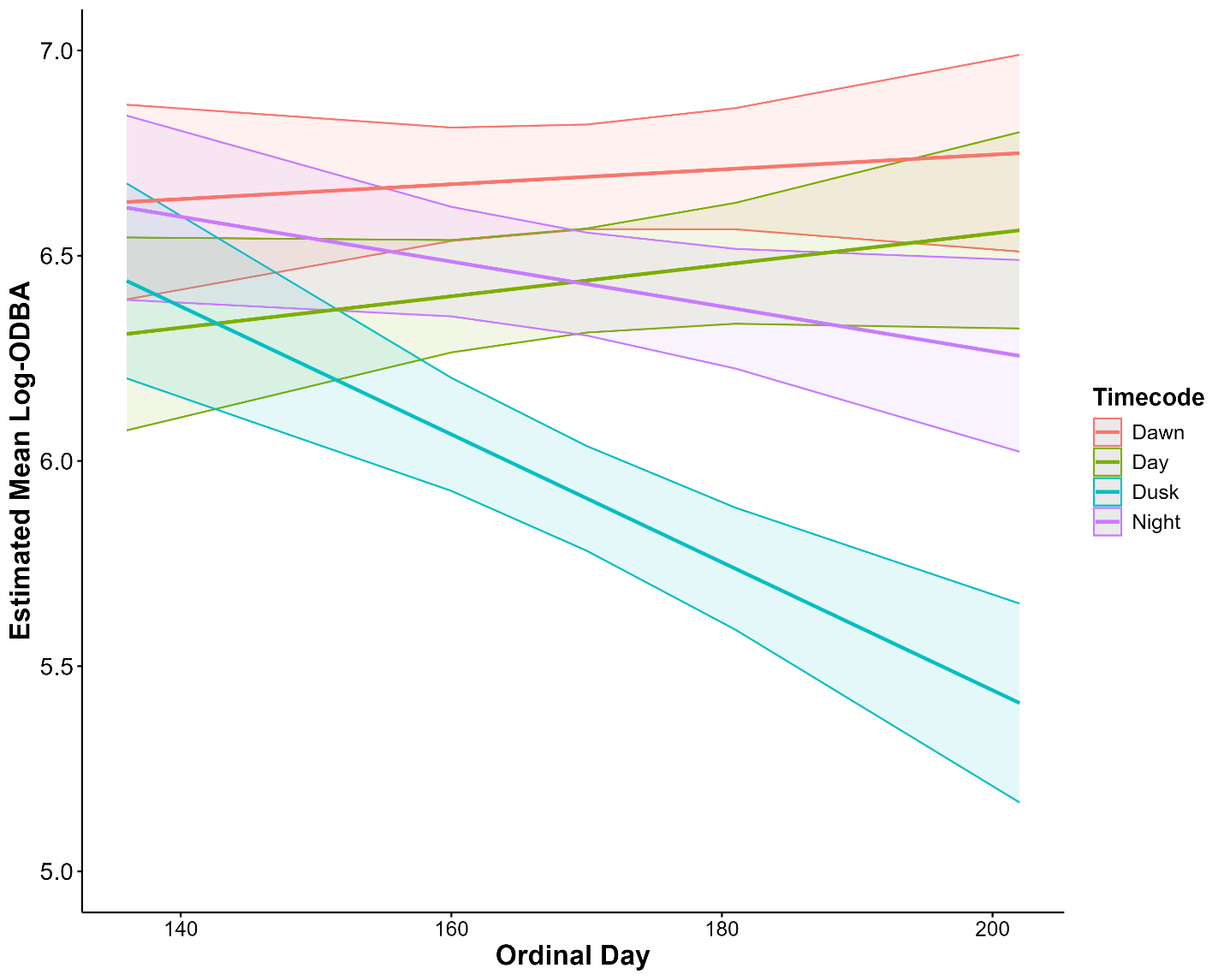
 Figure 1: Estimated mean overall dynamic body acceleration (ODBA) values collected from GPS/GSM transmitters deployed on breeding tagged black-crowned night herons (*Nycticorax nycticorax*). Dates span between the first date birds were tagged and when the last breeding bird left the colony. Breeding birds’ estimated average activity at dusk decreased over the reproductive period. The shaded region reflects the 95% confidence intervals for the estimate.


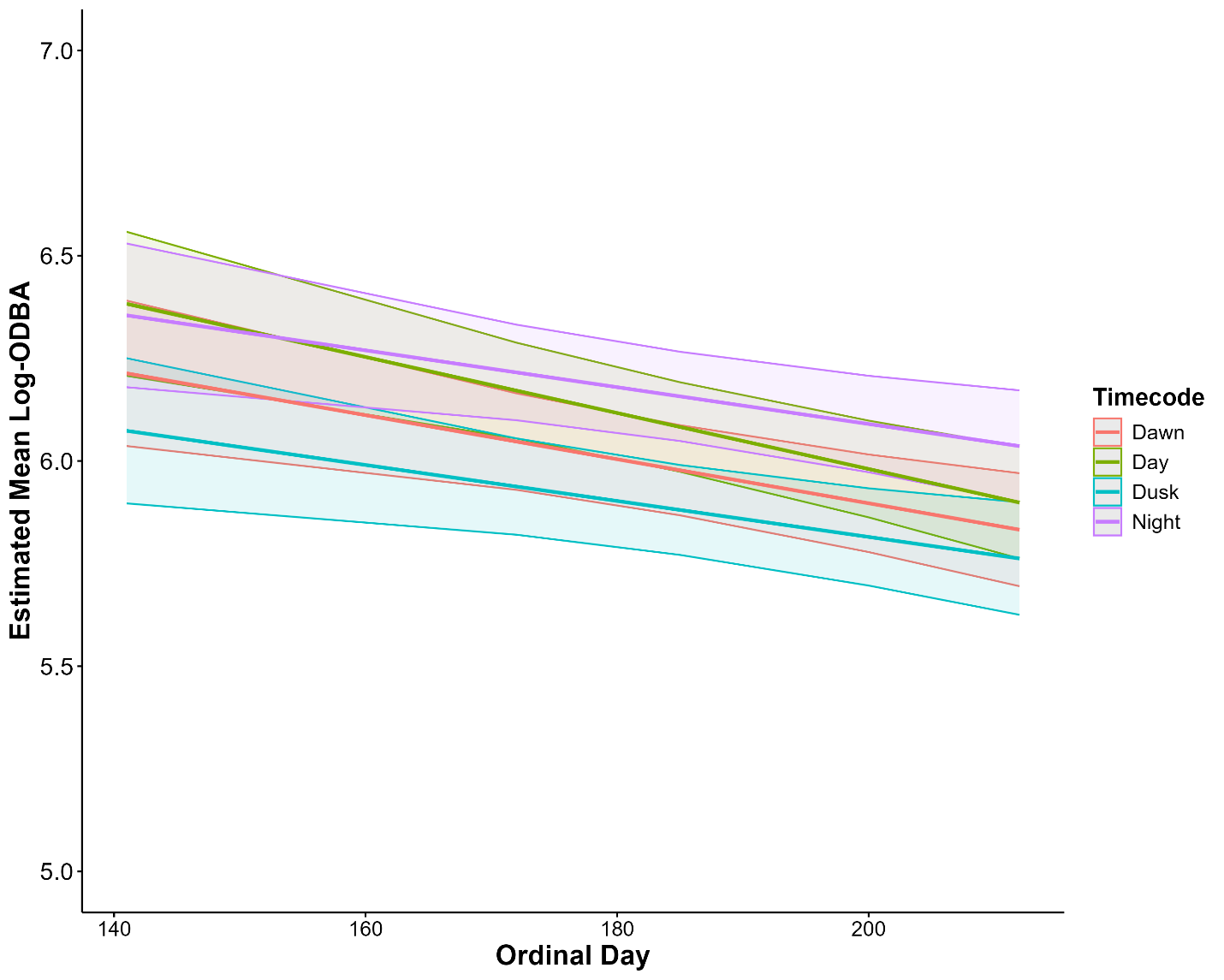
 Figure 2: Estimated mean overall dynamic body acceleration (ODBA) values collected from GPS/GSM transmitters deployed on non-breeding tagged black-crowned night herons (*Nycticorax nycticorax*). Dates span between the date the first tagged bird dispersed from the colony and the end of the reproductive period on 31 July 2024. As birds concluded breeding activities, their estimated mean ODBA decreased across all times of day. The shaded region reflects the 95% confidence intervals for the estimate.
